# Characterization of CT scans of patients with Birt-Hogg-Dubé syndrome compared with those of Chinese patients with non-BHD diffuse cyst lung diseases

**DOI:** 10.1186/s13023-020-01448-y

**Published:** 2020-07-06

**Authors:** Wenshuai Xu, Zhiyan Xu, Yaping Liu, Yongzhong Zhan, Xin Sui, Ruie Feng, Min Peng, Xue Li, Jun Wang, Shuzhen Meng, Li Wang, Xinlun Tian, Xue Zhang, Kai-Feng Xu

**Affiliations:** 1 Department of Pulmonary and Critical Care Medicine, Peking Union Medical College Hospital, Chinese Academy of Medical Sciences, Peking Union Medical College, Beijing, 100730 China; 2 Department of Internal Medicine, Peking Union Medical College Hospital, Chinese Academy of Medical Sciences, Peking Union Medical College, Beijing, 100730 China; 3 Department of Medical Genetics, School of Basic Medicine, Chinese Academy of Medical Sciences, Peking Union Medical College, Beijing, 100730 China; 4 Department of Respiratory and Critical Care Medicine, Southern Medical University, Nanfang Hospital, Guangzhou, China; 5 Department of Radiology, Peking Union Medical College Hospital, Chinese Academy of Medical Sciences, Peking Union Medical College, Beijing, 100730 China; 6 Department of Pathology, Peking Union Medical College Hospital, Chinese Academy of Medical Sciences, Peking Union Medical College, Beijing, 100730 China; 7 Department of Statistics, School of Basic Medicine, Chinese Academy of Medical Sciences, Peking Union Medical College, Beijing, 100730 China

**Keywords:** Birt-Hogg-Dubé syndrome, FLCN, Image, Diffuse cystic lung diseases, Radiology

## Abstract

**Background and objective:**

The purpose of this study was to create a practical CT-based algorithm to differentiate Birt-Hogg-Dubé (BHD) syndrome from other diffuse cystic lung diseases (DCLD).

**Methods:**

The study was a retrospective review of the CT images of 33 patients with BHD syndrome, 33 patients with LAM, and 23 patients with NBNL (non-BHD and non-LAM) among DCLD patients. On the basis of the data collected, the CT images were reviewed again to evaluate the characteristics (size, number, distribution, and morphology) of pulmonary cysts.

**Results:**

Lower lung-predominant cysts were more likely to be found in patients with BHD syndrome than in patients with LAM or in the NBNL DCLD group. In the axial distribution, 18 of 33 patients in BHD group had cysts that were predominantly near the mediastinum, and all the patients in the LAM and NBNL DCLD groups had diffuse cysts. The appearance of fusiform cysts was more easily observed in patients in the BHD group. In total, 58% patients in the BHD group had less than 50 lung cysts, while all patients in the non-BHD group had more than 50 lung cysts. The biggest cyst was located in the lower lobe in 28 of 33 patients in the BHD group, while 11 of 33 patients in LAM group and 10 patients in the NBNL DCLD group had the biggest cyst in the lower lobe.

**Conclusion:**

The pulmonary cysts in patients with BHD tended to be fusiform, less numerous and located predominantly in the lower lobe and near the mediastinum. These radiologic pulmonary features could assist physicians in differentiating BHD from other DCLDs.

## Introduction

Birt-Hogg-Dubé syndrome (BHD) is a rare autosomal dominant disorder manifested by multiple pulmonary cysts by recurrent pneumothorax, fibrofolliculomas, and renal tumors. BHD is caused by germline mutation in the *folliculin* (*FLCN*) gene on chromosome 17p11.2 [1], a tumor suppressor gene known to be involved in the signaling of mammalian target of rapamycin (mTOR). Reports and the characterization of BHD in Chinese individuals are rare [2].

Pulmonary cysts have been described in most patients with BHD, and pneumothorax has a 33–38% incidence to occur among them [3]. Lung involvement is often the earliest phenotypic manifestation to appear, and most affected patients are asymptomatic [4, 5]. Though previous research has demonstrated the difference between BHD and lymphangioleiomyomatosis (LAM) [6], BHD needs to be distinguished from other conditions associated with diffuse cysts lung diseases (DCLD), including LAM, Langerhans cell histiocytosis (LCH), and lymphocytic interstitial pneumonitis (LIP). Computed tomography (CT) gives us an intuitive manner to observe the cyst distribution and properties in the thorax. In BHDs, cysts are typically located in the lower lung regions, bilaterally. Some researchers have concluded that the chest CT findings of patients with BHD syndrome were multiple thin-walled pulmonary cysts of various sizes, predominantly distributed in subpleural regions of the lung [6–8]. However, no studies concluded the statistical significance of the thoracic CT findings of lung cysts characteristics in BHD compared with other DCLDs in China.

The purposes of this study were to quantify pulmonary cysts in CT images of patients with BHD and to identify the independent parameters to help us differentially diagnose BHD syndrome from non-BHD DCLDs, which will decrease the misdiagnosis and underdiagnosis of DCLD.

## Methods

### Study population

This single-center, retrospective, observational study received institutional review board approval. Patients’ confidentiality was strictly maintained. We searched the computerized medical record system for patients with diagnosis of DCLD at Peking Union Medical College Hospital from January 2014 to February 2019. Those patients who had pneumothorax and chylothorax on CT scans were excluded. Thirty-three cases of BHD syndrome, 33 cases of LAM, and 23 cases of other DCLD were enrolled in this study. Patients with BHD syndrome were all diagnosed by genetic testing. A total of 20 BHD patients were reported in our previous study [9], and the remaining were diagnosed from 2017 to 2019 [10]. Given the relative high incidence of LAM and to avoid the selection bias of the control group, we randomly selected 33 cases using a random number table method from 297 patients with definite LAM in the DCLD cohort at Peking Union Medical College Hospital during the same period as the selection of BHD patients. We selected all the non-BHD and non-LAM (NBNL) patients who received a definite diagnosis in the same period and ultimately recruited 23 NBNL DCLD patients. The diagnosis of LAM is based on the American Thoracic Society/Japanese Respiratory Society Clinical Practice Guideline [11]. Other etiologies of DCLDs include 15 cases of Sjogren’s syndrome (SS) related DCLD, 7 cases of pulmonary LCH (PLCH), 1 case of LCH and 1 case of Castleman’s Disease. All the patients with PLCH and Castleman’s Disease were diagnosed by lung biopsy. Patients with Sjogren’s syndrome were diagnosed by lung biopsy or the criteria for the classification of primary Sjogren’s syndrome published in the American College of Rheumatology (ACR) and the European League Against Rheumatism (EULAR) in 2016 [12].

### CT scan analysis of BHD & Control

For 52 of 89 patients (58%), high-resolution CT scans of the chest were available, with a slice thickness of 3 mm or less. For the remaining 37 patients (42%), CT scans were available, with a slice thickness of 4–5 mm. All CT images were evaluated by two pulmonologists (WX and ZX) and one radiologist separately (XS). Pulmonary cysts were defined as an air-filled space with a sharply demarcated thin wall. The total number of lung cysts in each patient was assessed as few if there were fewer than 10 cysts, several for 10–20, numerous for 20–50, or abundant if there were more than 50 cysts. This kind of stratification will change the continuous variables into categorical variables and decrease the uncertainty as much as possible. Cyst distribution was classified for both cranial-caudal distribution (as upper lung-predominant, lower lung-predominant or diffuse) and axial distribution (as near peripheral pleura, near mediastinum, or diffuse), and the predominance was defined as more than 50% of cysts [13]. The special fusiform shape of cysts was noted in all CT scans. The size and craniocaudal distribution of the biggest pulmonary cysts were recorded in both BHD and non-BHD patients.

### Statistical methods

Data were analyzed using SPSS for Windows version 24.0 (IBM Corp., USA) and are reported as the means (SDs) or medians (IQRs). The unpaired *t-*test or *Mann-Whitney U*-test was used to compare continuous variables. Categorical variables were compared using *Pearson’s chi-squared test*. The *Kruskal-Wallis test* was used for categorical variables to compare groups. For all analyses, two-sided tests and a significance level of 0.05 were used.

## Results

### Demographic features of all patients

Comparisons of the gender ratio, age of diagnosis, smoking history, pneumothorax and family history are listed in Table 1. All patients from the different families were of Chinese Han origin. Females were predominant in all groups. Among the patients with BHD, almost half of patients were misdiagnosed with primary spontaneous pneumothorax. The longest misdiagnosis duration for patients with BHD was 38 years. The average misdiagnosis delay for patients with BHD was 7.73 years. Eighteen (54.5%, 18/33) patients had cutaneous lesions. Renal impairment was observed in 11 (33%, 11/33) patients. Compared with the NBNL group, patients with BHD syndrome were more likely to have past medical histories of pneumothoraxes (*p* = 0.003), but there was no difference between BHD and LAM (BHD 17/33 [51.5%] vs LAM 12/33 [36.4%], *p* = 0.215). Significant difference was noted in family history of pneumothoraxes between the BHD group and either the LAM or the NBNL group. Smoking history had no significant difference between the BHD group and either the LAM or the NBNL group (Table 1).

**Table 1:** Demographic features of the BHD and non-BHD groups

Characteristic	BHD *N* = 33	LAM *N* = 33	NBNL DCLD^a^ *N* = 23	P*	P**
Female, N (%)	31 (93.9)	33 (100)	18 (78.3)	0.473	0.182
Age of diagnosis, Mean (SD)	48 ± 12	37 ± 8	40 ± 14	< 0.001	0.020
Pneumothorax antecedent, N (%)	17 (51.5)	12 (36.4)	3 (13.0)	0.215	0.003
Smoking history, N (%)	1 (3.0)	0 (0)	8 (34.8)	0.314	0.001
Family history, N (%)	25 (75.8)	0 (0)	0 (0)	< 0.001	< 0.001

*P values are for comparison between the BHD and LAM groups

**P values are for comparison between the BHD and NBNL DCLD groups

^a^The NBNL (no-BHD and no-LAM) DCLD group includes 15 cases of lymphocytic interstitial pneumonia associated with Sjogren’s syndrome (SS), 7 cases of pulmonary Langerhans cell histiocytosis (PLCH), 1 case with Langerhans cell histiocytosis (LCH) and 1 case of Castleman Disease

The *P* value was calculated with the use of the unpaired *t-test* to compare continuous variables

The *P* value was calculated with the use of *Pearson’s chi-squared test* for categorical variables

*Abbreviations*: *BHD* Birt-Hogg-Dubé syndrome, *DCLD* diffuse cystic lung disease, *NBNL* no-BHD and no-LAM, *SD* standard deviation

### Radiological features of pulmonary cysts in BHD and non-BHD

**Fig. 1:**
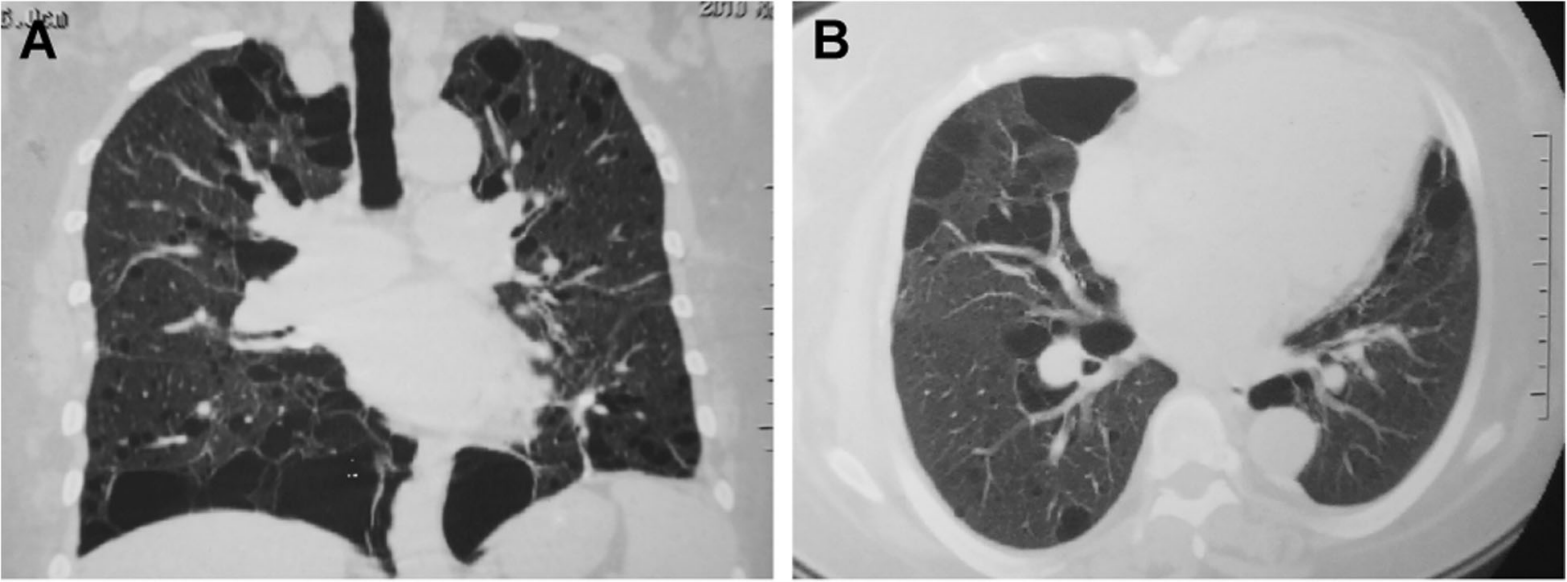
Thoracic CT findings of patients with BHD. **a** Coronal reformatted image of high-resolution chest CT images showing lower lung predominant, para-mediastinal, and fusiform cysts in BHDs; **b** Axial high-resolution chest CT images through the lower lungs

**Fig. 2:**
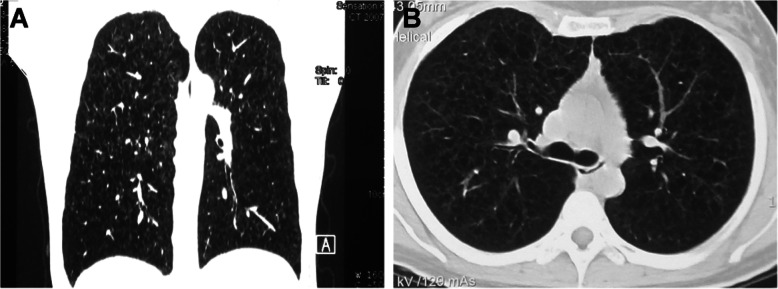
Thoracic CT findings of patients with LAM. **a** Coronal reformatted image of high-resolution chest CT images in a patient with LAM shows diffuse cysts; **b** Axial high-resolution chest CT images through the upper lungs

Cysts were bilateral in all patients. Lower lung-predominant cysts were more likely to be found in patients with BHD syndrome (58% of patients) (Fig. 1) than in patients in the LAM (no patients, *p* < 0.001) (Fig. 2) or the NBNL group (26% of patients, *p* = 0.020). Significant difference was also found in the axial distribution of pulmonary cysts. All cysts were diffuse in the non-BHD group, and 18 of 33 patients (55%) in the BHD group had cysts predominance near the mediastinum (*p* < 0.001).

The morphology of the pulmonary cysts was variable within individual patients. The appearance of fusiform cysts was observed in 27 patients in the BHD group (82%), while only 5 cases had fusiform cysts in the LAM group (15%, *p* < 0.001) and 5 patients in the NBNL group (22%, *p* < 0.001).

In terms of the number of cysts, 58% of patients in the BHD group had fewer than 50 lung cysts, while all patients in the LAM group (*p* < 0.001) and 78% patients in the NBNL group had more than 50 lung cysts (*p* = 0.008).

**Fig. 3:**
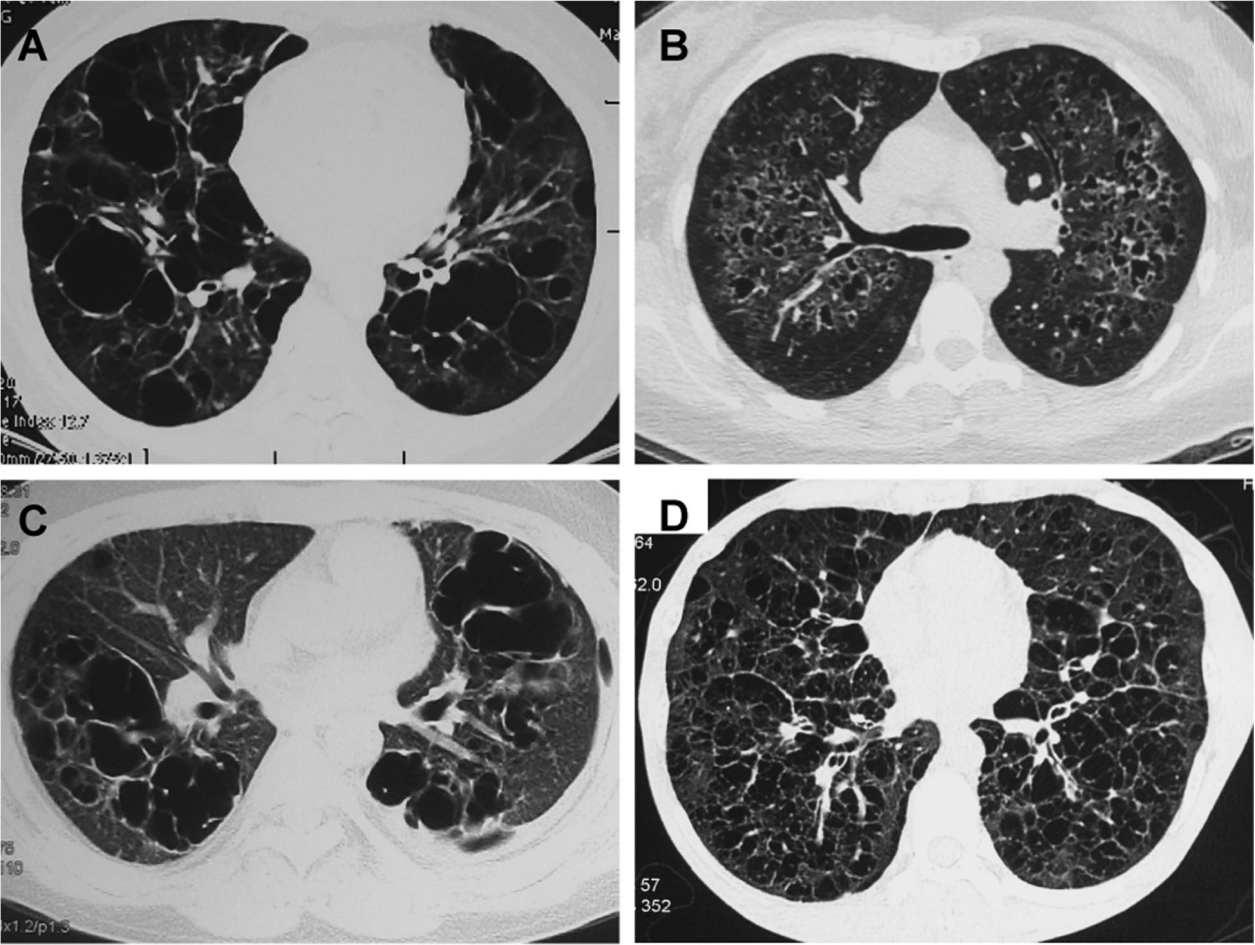
Thoracic CT findings of patients with non-BHD and non-LAM (NBNL) DCLDs. **a** CT images of patients with Sjogren’s syndrome; **b** CT images of patients with Pulmonary Langerhans Cell Histiocytosis; **c** CT images of patients with Langerhans Cell Histiocytosis; **d** CT images of patients with Castleman’s disease

The distribution of the largest cysts was different. The largest cyst was located in the lower lobe in 28 patients (85%) in the BHD group, while 11 of 33 patients (33%) in the LAM group and 10 patients (43%) in the NBNL group had the largest cyst in the lower lobe. When comparing the diameters of the largest cysts between patients in two groups, the median diameters were 45 mm in the BHD group and 16.5 mm in the LAM group. Significant differences in the distribution of the diameter of the largest cysts were noted between the BHD and LAM groups (*p* < 0.001) and the NBNL group (*p* = 0.001) (Table 2 and Fig. 3).

**Table 2:** Radiological characteristics of pulmonary cysts in BHD and non-BHD

Characteristic	BHD *N* = 33	LAM *N* = 33	NBNL DCLD *N* = 23	P*	P**	P***
**Cranial-caudal distribution**						
Upper lung, N (%)	2 (6)	0 (0)	6 (26)	< 0.001	0.473	0.086
Lower lung, N (%)	19 (58)	0 (0)	6 (26)	< 0.001	< 0.001	0.020
Diffuse, N (%)	12 (36)	33 (100)	11 (48)	< 0.001	< 0.001	0.391
**Axial distribution**						
Mediastinum, N (%)	18 (55)	0 (0)	0 (0)	< 0.001	< 0.001	< 0.001
Peripheral pleural, N (%)	3 (9)	0 (0)	0 (0)	0.074	0.237	0.237
Diffuse, N (%)	12 (36)	33 (100)	23 (100)	< 0.001	< 0.001	< 0.001
**Fusiform Cysts,** N (%)	27 (82)	5 (15)	5 (22)	< 0.001	< 0.001	< 0.001
**No. of Lung Cysts**						
<10, N (%)	2 (6)	0 (0)	0 (0)	0.180	0.473	0.638
10–20, N (%)	4 (12)	0 (0)	0 (0)	0.030	0.122	0.228
20–50, N (%)	13 (39)	0 (0)	5 (22)	< 0.001	< 0.001	0.164
>50, N (%)	14 (42)	33 (100)	18 (78)	< 0.001	< 0.001	0.008
**Maximum Cyst**						
Lower lung, N (%)	28 (85)	11 (33)	10 (43)	0.023	< 0.001	0.001
Bigger cyst diameter, median (IQR)	45 (30, 55)	16.5 (10,20)	35 (20,50)	< 0.001	< 0.001	0.121

P*values are for comparison within the three groups together by *Kruskal-Wallis test*

P**values are for comparison between the BHD and LAM groups

P*** values are for comparison between the BHD and NBNL DCLD groups

The *P* value was calculated with the use of *Pearson’s chi-squared test* for categorical variables

The *P* value was calculated with the use of the *Wilcoxon rank-sum test* to compare continuous variables

## Discussion

This is a retrospective study focusing on the thoracic CT findings of lung cysts in BHD compared with those in Chinese patients with other DCLDs. We used quantification methods to confirm that the pulmonary cysts in patients with BHD tend to be fusiform, less numerous and located predominantly in the lower lobe and near the mediastinum compared with cysts in patients with other types of DCLD.

BHD has often been confused with other kinds of DCLD in the clinic, not only because of its low prevalence, but its low awareness. Most of our BHD patients were referred to our hospital due to the suspicion of other kinds of DCLD, such as LAM, since our clinic is the largest LAM/TSC center in China. Extrapulmonary abnormalities, including kidney and skin manifestations, are rare in Chinese individuals [9, 10] compared with Caucasians, and respiratory symptoms may be the only symptom observed in Chinese BHD patients. Therefore, it is important to recognize the radiologic features of the lung in BHD and to differentiate these features from those of other diffuse cystic lung diseases. Some researchers believed that pulmonary cysts were predominantly localized in the medial and lower zones in BHD compared with other DCLDs, the majority of which were based on experiences from specialists [14–16].

Abundant studies focused on the descriptive characteristics of pulmonary cysts of BHD, whereas statistical comparisons of the lung cysts between BHD and other DCLDs have been rare. Tobino and colleagues performed a quantitative analysis of pulmonary cysts on CT between BHD and LAM [6]. They found that compared with patients with LAM, the cysts in BHD patients had a more irregular shape, more septation, lower and more peripheral distribution, larger maximum size, and more attachment to the pleura. It is not very difficult to differentiate BHD from LAM, as the latter has more homogeneous cysts compared with BHD. Whether the characteristics of pulmonary images of BHD will be different from those of NBNL patients has not yet been determined. In our study, for the first time, to the best of our knowledge, we are trying to distinguish BHD from other kinds of NBNL groups based on digitalized radiologic pulmonary features. We verified the speculation from a previous study [6] that the cysts in BHD patients had a more irregular shape, more septation, lower and more mediastinum distribution, and larger maximum size compared with LAM. Meanwhile, we also found that NBNL DCLDs have similar morphology and distribution and have less fusiform, peripheral, and para-mediastinal cysts. Those findings are consistent with the prior study, which reported that compared with patients with LIP or LAM, patients with BHD syndrome were significantly more likely to have elliptical (floppy) para-mediastinal cysts or a disproportionate number of para-mediastinal cysts [17]. Our study also found that compared with patients with NBNL DCLDs or LAM, patients with BHD syndrome have fewer cysts, as the number of cysts in half of the patients ranges from 20 to 50, and the largest cyst is often located in the lower lobe. Therefore, pulmonary cysts in BHD have specific radiologic features that can be different from other kinds of DCLDs. However, we found that BHD and NBNL DCLDs have similar diameters of the largest cysts, and both are larger than the diameters of the largest cysts in LAM. As a result, the diameters of the largest cyst could not be used to distinguish BHD from NBNL DCLDs.

The pathogenesis of lung cysts distribution is still unknown. Some hypotheses have explained the pathogenesis based on the effect of the mutation in FLCN on the epithelial layer on the inside of the pleural cysts. The downregulation of folliculin was followed by increased cell-cell adhesion [18]. It is much more likely that the development and recurrence of pneumothorax in BHD is related to the lack of epithelial layers to stretch, but a therapeutic study is needed [19].

Our study may have several limitations. First, the number of patients with BHD included is relatively small. Second, three-dimensional reconstruction of lung cysts is hard to achieve, and the number and distribution of cysts were observed in the horizontal direction, causing some deviation in the data collection. However, two professional respiratory physicians and one radiologist worked independently and in random order to assess the thoracic CT results in patients to decrease any possible counting deviations.

## Conclusion

In conclusion, our study provides evidence that fusiform para-mediastinal cysts of various sizes show bilateral lower lung zone predominance and that a number of lung cysts less than 50 is a characteristic CT finding that distinguishes BHD from other cystic lung diseases, making it possible to perform a detailed evaluation of pulmonary cysts on thoracic thin-section CT. Molecular analysis of the FLCN gene should be systematically conducted in patients with cystic lung diseases in such cases.

## Data Availability

The datasets used and analysed during the current study area available from the corresponding author.
